# Association between plasma leptin/adiponectin ratio and insulin resistance indexes in prepubertal children

**DOI:** 10.20945/2359-4292-2022-0353

**Published:** 2024-01-29

**Authors:** Carolina Bravo, Verónica Mericq, Ana Pereira, Camila Corvalán, Hugo E. Tobar, José Patricio Miranda, José Luis Santos

**Affiliations:** 1 Pontificia Universidad Católica de Chile Facultad de Medicina Departamento de Nutrición, Diabetes y Metabolismo Santiago Chile Departamento de Nutrición, Diabetes y Metabolismo, Facultad de Medicina, Pontificia Universidad Católica de Chile, Santiago, Chile; 2 Universidad de Chile Instituto de Nutrición y Tecnología de Alimentos Santiago Chile Instituto de Nutrición y Tecnología de Alimentos, Universidad de Chile, Santiago, Chile; 3 Universidad de Chile Facultad de Medicina Instituto de Investigaciones Materno-Infantil Santiago Chile Instituto de Investigaciones Materno-Infantil, Facultad de Medicina, Universidad de Chile, Santiago, Chile

**Keywords:** Insulin resistance, leptin, adiponectin, leptin/adiponectin ratio

## Abstract

**Objective:**

To assess the association between leptin/adiponectin ratio (LAR) and insulin resistance surrogates in prepubertal children.

**Subjects and methods:**

Study based on data from the Growth and Obesity Chilean Cohort Study (GOCS) involving 968 Chilean prepubertal children. Plasma insulin, leptin, and adiponectin were determined by immunoassays. Several common insulin resistance surrogates were calculated, including the homeostasis model assessment of insulin resistance (HOMA-IR), triglyceride/HDL cholesterol index, triglyceride-glucose (TyG) index, and the TyG index corrected for body mass index (BMI; TyG-BMI) and waist circumference (WC; TyG-WC). Associations among variables were assessed using multiple linear and logistic regression analysis.

**Results:**

There was a significant direct association between plasma leptin and LAR with BMI z-score but no association between plasma adiponectin and adiposity. After adjustments for sex and age, LAR was significantly associated with all insulin resistance surrogates (which were categorized using the 75th percentile as the cutoff point), with the TyG-WC index emerging as the surrogate with the highest magnitude of association (odds ratio [OR] 2.44, 95% confidence interval [CI] 2.05-2.9). After additional adjustment for BMI z-score, only the association between LAR and TyG-WC remained significant (OR 1.64, 95% CI 1.27-2.12).

**Conclusion:**

Plasma leptin and LAR were strongly associated with several common insulin resistance surrogates in prepubertal children, most notably with the TyG-WC index. Associations between LAR and insulin resistance indexes were mainly driven by the effect of plasma leptin, which is also directly associated with increased adiposity.

## INTRODUCTION

The adipose tissue secretes adipokines like leptin and adiponectin, which play essential physiological roles (1). Circulating leptin is directly associated with body fat and is involved in appetite regulation, energy homeostasis, and obesity-related proinflammatory status (2). Plasma leptin concentrations and adiposity have also been associated with type 2 diabetes mellitus and metabolic syndrome (3). On the other hand, patients with leptin deficiency due to mutations in the leptin gene, a rare genetic disease, are morbidly obese and present with hyperphagia from infancy (4). In these patients, exogenous leptin leads to weight loss and enhanced insulin sensitivity (5). However, in multifactorial obesity, exogenous leptin administration does not improve insulin sensitivity, possibly due to leptin resistance. Adiponectin, one of the most abundant serum hormones (6), has been associated with anti-inflammatory and insulin-sensitizing effects (1). Interestingly, congenital generalized lipodystrophy is characterized by very low levels of circulating adipokines, extreme leanness, severe insulin resistance, and early-onset diabetes. This illustrates the fact that the complex relationships between adiposity, plasma leptin, plasma adiponectin, and insulin resistance lie beyond obesity. In these patients with lipodystrophy, the administration of exogenous leptin enhances insulin sensitivity and induces appreciable reductions in plasma glucose and triglycerides (5).

Insulin resistance is becoming a serious problem in adults and children (7) due to the increased prevalence of childhood overweight/obesity (8). In this sense, cross-sectional studies in the prepubertal stage have shown higher fasting insulin concentrations in girls compared with boys (9,10). Moreover, there is a physiologic increase in plasma insulin and insulin resistance in puberty, which extends to adolescence in the presence of overweight/obesity (11). Several insulin resistance surrogates based on fasting plasma samples have been extensively used in epidemiological studies, such as the homeostasis model assessment of insulin resistance (HOMA-IR) index and the plasma leptin/adiponectin ratio (LAR). These indexes show a relatively good correlation with gold-standard reference methods (12), such as those derived from the oral glucose tolerance test, intravenous glucose tolerance test, and hyperinsulinemic-euglycemic clamp (13,14). In children, several surrogates of insulin resistance have also been used (15), including the HOMA-IR (16), the plasma triglyceride-glucose index (TyG index) and TyG index corrected for body mass index (BMI; TyG-BMI index) and waist circumference (WC; TyG-WC index) (17,18), and the plasma triglyceride to HDL cholesterol (TG/HDL) ratio (1,19–22). However, few studies have evaluated the performance of LAR as an insulin resistance biomarker in children, especially in the prepubertal age (1,23,24). Thus, the aim of this study was to assess the association between plasma LAR and insulin resistance surrogates in prepubertal children.

## SUBJECTS AND METHODS

### Study design and subjects

The present study is based on data from the Growth and Obesity Chilean Cohort Study (GOCS) (25–27). Briefly, the GOCS is a population-based cohort study that was initiated in 2006 and included Chilean children born in 2002-2003 (aged 2.6 to 4 years at the time of recruitment). The participants were selected from the National Nursery Schools Council Program of six neighborhoods in Santiago (Chile), a representative group of Chilean children from low- to middle-income households. Inclusion in the cohort was restricted to children born at term from singleton gestations, with birth weight between 2,500 and 4,500 grams and no evidence of diseases at birth or at the time of recruitment. After agreement with parents, a total of 1,195 participants were finally selected from 1,498 eligible children (80%); more details of the cohort have been published elsewhere (28). In the present study, we conducted a cross-sectional analysis of 968 prepubertal children (Tanner stage 1) evaluated in the year 2009 (mean age 6.8 ± 0.4 years; 48% girls) who had a complete set of biochemical and anthropometric measurements. Written informed consent was obtained from the parents, and written assent was obtained from the children. The study was approved by the Ethics Committee of the Institute of Nutrition and Food Technology (INTA) at the University of Chile (Reference: 1090252).

### Anthropomtric measurements, pubertal classification, and nutritional status

Anthropometric measurements were carried out using standardized techniques by trained personnel. Weight was measured using a Tanita BC-418 device (Tanita Corporation of America, Inc., Arlington Heights, IL, USA) with a precision of 0.1 kg. Height was measured using a wall-mounted Harpenden stadiometer (Holtain, Crosswell, Wales) to the nearest 0.1 cm. The WC, defined as the minimum circumference between the iliac crest and the rib cage, was measured using an inextensible metal tape (model W606PM; Lufkin) to the closest 0.1 cm. The intra-observer technical error of measurement and the mean average bias of the observer were within the limits suggested in the WHO Multicentre Growth Reference Study (29). Measures of weight, height, and WC were used to calculate the BMI and waist-to-height ratio (WHR). The WHO 2007 growth reference curve was used to calculate BMI z-scores through scripts available in the WHO AnthroPlus software (30). All children were classified as Tanner stage 1 based on secondary sexual characteristics of external genitalia, *i.e.*, phallus, scrotum, and testes volume in boys; breasts in girls; and pubic hair in both boys and girls (31). This evaluation was performed by two dietitians trained by a pediatric endocrinologist (one female dietitian assessed all girls, and one male dietitian assessed all boys). Values of BMI were categorized according to z-scores as normal weight (between −2 and 1 standard deviations [SDs]), overweight (from ≥ 1 SD to ≤ 2 SDs), and obesity (≥ 2 SDs).

### Biochemical evaluations

Fasting blood samples were drawn into EDTA tubes. Plasma glucose was measured by a colorimetric method (GOD-PAP commercial kit *Química Clínica Aplicada SA*). Plasma insulin levels (μU/mL) were measured by radioimmunoassay (RIA; sensitivity 0.4 μU/mL, intra-assay coefficient of variation [CV] 5.2, inter-assay CV 7.3). Plasma adiponectin was measured by RIA (Human Adiponectin RIA Kit; Linco Research, Inc.; St. Charles, MO, USA; sensitivity 1.0 ng/mL, intra-assay CV 3.86, inter-assay CV 8.46). Plasma leptin was also measured by RIA (Human Leptin RIA Kit; Linco Research, Inc.; sensitivity 1.0 ng/mL, intra-assay CV 4.98, inter-assay CV 4.5). Lipid profile (plasma triglycerides, HDL cholesterol, and total cholesterol) was determined using dry analytic methodology (VITROS, Ortho Clinical Diagnostics, Inc., Raritan, NJ, USA). All biochemical analyses in plasma were carried out at the Nutrition Laboratory of the *Pontificia Universidad Católica de Chile* (26).

### Insulin resistance indexes

The insulin resistance indexes were based on fasting plasma samples and biochemical measurements described in the above paragraph and were calculated as follows (5,32–34):

- HOMA-IR = [fasting insulin (μU/mL) X fasting glucose (mg/dL)]/405;
- TyG index = Ln [fasting triglycerides (mg/dL) x fasting plasma glucose (mg/dL)]/2;
- TyG-BMI = TyG index x BMI (kg/m^2^);
- TyG-WC = TyG index x waist circumference (cm);
- TG/HDL ratio = plasma triglyceride (mg/dL) ÷ HDL cholesterol (mg/dL);
- LAR = plasma leptin (ng/mL) ÷ plasma adiponectin (μg/mL).

### Statistical analyses

Summary statistics were presented as averages (±SD), medians (and percentiles), or proportions, depending on the nature of the variable. Differences by sex in biochemical variables, anthropometric measures, and insulin resistance surrogates were evaluated using multiple linear regression. Subsequently, multiple linear regression and logistic regression techniques were used to assess associations between plasma adipokines – leptin, adiponectin, and LAR – as independent variables and BMI z-score categories or insulin resistance indexes as dependent variables using models involving different sets of dependent and independent variables, as well as covariates. For the logistic models, insulin resistance (dependent variable) was defined as a HOMA-IR, TyG index, TyG-BMI index, TyG-WC index, or TG/HDL ratio above the 75th percentile. In these analyses, the independent variables plasma leptin, adiponectin, and LAR were standardized (mean = 0; SD = 1) using a rank-based inverse-normal function. Logistic regression analyses were adjusted by sex and age (model 1) and sex, age, and BMI z-score (model 2).

Receiver operating characteristic (ROC) curve analysis was used to evaluate the discriminatory capacity of LAR, leptin, adiponectin, and BMI z-score in classifying children as insulin resistant or sensitive, defined by the 75th percentile of different insulin resistance surrogates. A comparison of ROC curves was carried out by the tests of equality of ROC curve areas.

All statistical analyses were carried out with the use of Stata software, version 17.0 (StataCorp LLC, College Station, TX, USA), with a significance threshold for p set at 0.05.

## RESULTS

Table 1 summarizes the clinical, anthropometric, and biochemical characteristics of the participants in the present study. Using linear regression analysis, we observed between girls and boys significant differences in fasting glycemia, plasma LDL cholesterol, and total cholesterol but no significant differences in the other variables. Table 2 shows different surrogates of insulin resistance (HOMA-IR, TG/HDL ratio, TyG index, TyG-BMI index, TyG-WC, and LAR), indicating no notable differences between girls and boys. The prevalence of obesity, defined as a BMI z-score ≥ 2, was 13.1% in girls (95% confidence interval [CI] 10.2-16.5%) and 20.8% in boys (95% CI 10.2-16.5%).

**Table 1:** Anthropometric and plasma biochemical measurements of Chilean prepubertal children

	Mean	SD			Percentiles		
			10	25	50	75	90
**GIRLS (n = 465)**							
Age (years)	6.8	0.4	6.1	6.5	6.8	7.1	7.3
Height z-score	0.2	0.9	-0.9	-0.5	0.2	0.8	1.4
Weight (kg)	25.0	4.5	19.9	21.7	23.8	27	31.4
BMI z-score	0.8	1.0	-0.5	0.1	0.7	1.5	2.3
Waist circumference	58.6	6.3	51.5	54.1	57.5	62.0	68.1
Waist-to-height ratio	0.49	0.05	0.43	0.46	0.48	0.51	0.55
Fasting plasma glucose (mg/dL)	88.9*	6.4	82	85	89	92	97
Fasting plasma insulin (μU/mL)	5.6	1.6	4.6	4.9	5.2	5.7	6.5
Total cholesterol (mg/dL)	168.5*	25.6	136	150	168	185	201
Triglycerides (mg/dL)	94.1	39.9	53	66	85	112	148
LDL cholesterol (mg/dL)	99.4*	24.9	68.8	83	98.6	114.6	132.4
HDL cholesterol (mg/dL)	50.3	12.4	35	42	50	58	65
Plasma leptin (ng/mL)	5.9	4.1	2.4	3.3	4.7	7.2	11.6
Plasma adiponectin (μg/mL)	17.7	6.3	10.3	13.1	16.8	21.2	25.8
**BOYS (n = 503)**							
Age (years)	6.8	0.5	6.1	6.5	6.9	7.1	7.3
Height z-score	0.1	0.9	-1.1	-0.5	0.1	0.8	1.4
Weight (kg)	25.4	4.9	19.9	21.7	24.5	28	32.2
BMI z-score	0.9	1.3	-0.5	0.02	0.8	1.8	2.6
Waist circumference	59.0	6.6	52.0	54.2	57.4	62.2	68.3
Waist-to-height ratio	0.49	0.05	0.44	0.45	0.48	0.51	0.55
Fasting plasma glucose (mg/dL)	90.3*	6.3	83	86	91	94	98
Fasting plasma insulin (μU/mL)	5.4	1.3	4.5	4.9	5.1	5.5	6
Total cholesterol (mg/dL)	165.2*	27.5	132	146	164	184	200
Triglycerides (mg/dL)	94.3	46.1	48	61	80	119	150
LDL cholesterol (mg/dL)	95.7*	27.9	60.4	78.4	96.8	112	130.6
HDL cholesterol (mg/dL)	50.6	14.8	35	40	49	58	68
Plasma leptin (ng/mL)	5.7	3.8	2.4	3.3	4.6	6.8	10.5
Plasma adiponectin (μg/mL)	18.3	7.2	9.9	13.2	17.6	22.3	27.8

* Significant differences between girls and boys evaluated by multiple regression.

**Table 2:** Surrogate indexes of insulin resistance in Chilean prepubertal children

	Mean	SD			Percentiles		
			10	25	50	75	90
**GIRLS (n = 465)**							
HOMA-IR	1.2	0.4	1.0	1.1	1.1	1.3	1.5
TG/HDL ratio	2.0	1.2	0.9	1.3	1.8	2.4	3.4
TyG index	4.5	0.2	4.2	4.3	4.5	4.6	4.7
TyG-BMI	76.7	11.6	63.8	68.4	74.4	82.6	93.6
TyG-WC	262.5	33.3	225.0	239.0	256.0	283.3	310.4
LAR	0.4	0.3	0.1	0.2	0.3	0.5	0.8
**BOYS (n = 503)**							
HOMA-IR	1.2	0.3	1.0	1.1	1.1	1.3	1.4
TG/HDL ratio	2.1	1.3	0.8	1.2	1.8	2.6	3.8
TyG index	4.5	0.2	4.2	4.3	4.5	4.6	4.8
TyG-BMI	77	12.2	64.5	68.2	74.7	83.3	94.1
TyG-WC	264.1	35.6	225.8	239.7	257.6	279.8	314.5
LAR	0.4	0.4	0.1	0.2	0.3	0.4	0.7

Abbreviations: HOMA-IR, homeostasis model assessment of insulin resistance; TG/HDL ratio, triglyceride/HDL cholesterol ratio; TyG index, triglyceride-glucose index; TyG-BMI, triglyceride-glucose index corrected for body mass index; TyG-WC, triglyceride-glucose index corrected for waist circumference; LAR, plasma leptin/adiponectin ratio.

### Association between anthropometric variables and plasma adipokines (leptin, adiponectin, and leptin/adiponectin ratio)

**Figure 1:**
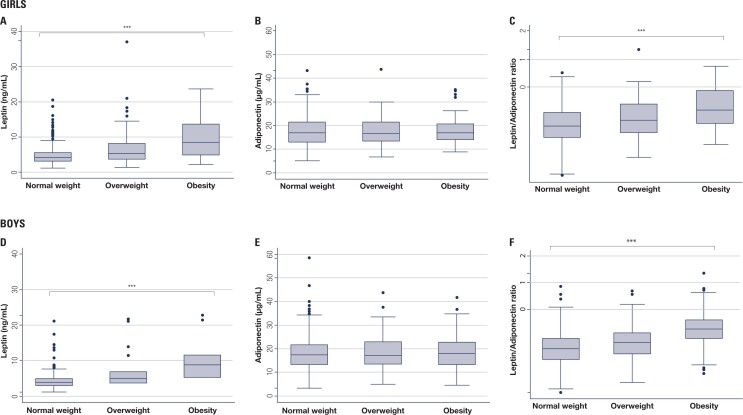
Plasma leptin, adiponectin, and leptin/adiponectin ratio (LAR) according to body mass index (BMI) categories in prepubertal Chilean children. Linear regression analysis of the association between plasma leptin, plasma adiponectin, and LAR with BMI z-score categories adjusted for age in girls (**A**, **B**, and **C**) and boys (**D**, **E**, and **F**). *** P value < 0.0001.

Linear regression analysis of the association between plasma leptin, plasma adiponectin, and LAR with BMI z-score categories adjusted for age was performed separately in girls and boys. In girls (278 with normal weight, 126 with overweight, and 61 with obesity), from normal weight to obesity, plasma leptin and LAR increased significantly (p < 0.001) (Figures 1A and 1C), while plasma adiponectin decreased without significant differences (p = 0.483) (Figure 1B). In boys (280 with normal weight, 118 with overweight, and 105 with obesity), from normal weight to obesity, plasma leptin and LAR also increased significantly (p < 0.001) (Figures 1D and 1F), while differences in plasma adiponectin were not significant (p = 0.368).

We also performed linear regression analyses to evaluate the association between plasma leptin, plasma adiponectin, and LAR with WHR categories defined by tertiles and adjusted for age in girls and boys separately. In girls, plasma leptin and LAR increased significantly from the lowest to highest WHR tertile (p < 0.001), while plasma adiponectin decreased with no significant differences (p = 0.37). In boys, plasma leptin and LAR also increased significantly from the lowest to the highest WHR tertile (p < 0.001). In contrast, differences in plasma adiponectin did not achieve statistical significance by WHR in boys (p = 0.826). Similar statistical associations and identical conclusions were achieved after using tertiles of WC instead of WHR in boys. However, in girls there were no significant associations between WC and plasma leptin, LAR, or adiponectin (data not shown). There were no significant differences in WC and WHR values between boys and girls.

We also calculated the odds ratios (ORs) for the association between WHR (as a dependent variable in separate models) and plasma leptin, plasma adiponectin, and LAR (as independent variables in separate models). The WHR was defined by using the 75th percentile as a cutoff point. Statistical models were adjusted for sex and age (model 1) and sex, age, and BMI z-score (model 2). In model 1, plasma leptin (OR 2.42, 95% CI 2.04-2.86) and LAR (OR 1.94, 95% CI 1.66-2.27) were both significantly associated with WHR.

### Association between plasma adipokines (leptin, adiponectin, and leptin/adiponectin ratio) and insulin resistance surrogates

Table 3 shows the ORs for the associations between plasma leptin, plasma adiponectin, and LAR (as independent variables in separate models) and insulin resistance indexes (as dependent variables in separate models). Insulin resistance was defined via different surrogates (HOMA-IR, TyG index, TyG-WC index, TyG-BMI index, or TG/HDL ratio) using the 75th percentile as a cutoff point. Statistical models were adjusted for sex and age (model 1) and sex, age, and BMI z-score (model 2). In model 1, plasma leptin and LAR were both significantly associated with all insulin resistance surrogates. Notably, plasma leptin was associated with the TyG-WC index (OR 3.04, 95% CI 2.51-3.68) (Table 3). When adjustment for BMI was included (model 2), plasma leptin was still significantly associated with TyG-WC (OR 1.71, 95% CI 1.31-2.22) (Table 3). The LAR followed a similar pattern of association (model 1: OR 2.44, 95% CI 2.05-2.90; model 2: OR 1.64, 95% CI 1.27-2.12) (Table 3). No significant associations were found between plasma adiponectin and insulin resistance indexes. Given that plasma adipokines and LAR were normalized to a mean of 0 and SD of 1, ORs in Table 3 are expressed as change in odds by 1 SD increment in leptin, adiponectin, or LAR.

**Table 3:** Odds ratios for the association of plasma leptin, plasma adiponectin, and leptin/adiponectin ratio with insulin resistance in Chilean prepubertal children

Insulin resistance indexes**		Leptin (ng/mL)	Adiponectin (μg/mL)	LAR
		OR (95% CI)	OR (95% CI)	OR (95% CI)
HOMA-IR	Model 1	**1.68 (1.35 to 2.09)**	1.08 (0.88 to 1.34)	**1.50 (1.21 to 1.85)**
	Model 2	1.12 (0.95 to 1.31)	1.09 (0.94 to 1.27)	1.04 (0.89 to 1.22)
TyG index	Model 1	**1.30 (1.12 to 1.51)**	1.02 (0.88 to 1.17)	**1.23 (1.06 to 1.42)**
	Model 2	1.14 (0.97 to 1.34)	1.02 (0.88 to 1.18)	1.09 (0.93 to 1.27)
TyG-WC	Model 1	**3.04 (2.51 to 3.68)**	0.98 (0.85 to 1.14)	**2.44 (2.05 to 2.90)**
	Model 2	**1.71 (1.31 to 2.22)**	0.89 (0.70 to 1.13)	**1.64 (1.27 to 2.12)**
TyG-BMI*	Model 1	**2.85 (2.37 to 3.43)**	1.01 (0.87 to 1.16)	**2.30 (1.94 to 2.73)**
TG/HDL	Model 1	**1.26 (1.09 to 1.46)**	1.05 (0.91 to 1.22)	**1.18 (1.02 to 1.36)**
	Model 2	1.13 (0.96 to 1.32)	1.06 (0.92 to 1.23)	1.05 (0.90 to 1.23)

Logistic regression models were used to assess associations between leptin, adiponectin, and leptin/adiponectin ratio (LAR) with surrogates of insulin resistance.

** Defined as above the 75th percentile of each insulin resistance surrogate (combined sample of girls and boys). Values in bold indicate statistical significance (p < 0.05).

Odds ratios per 1 standard deviation increment in plasma leptin, plasma adiponectin, or LAR are shown.

Model 1 was adjusted for sex and age; Model 2 was adjusted for sex, age, and body mass index (BMI) z-score.

* Models involving TyG-BMI were not adjusted by BMI z-score, given that BMI is already included in the definition of the TyG-BMI index.

Abbreviations: HOMA-IR, homeostasis model assessment of insulin resistance; TyG index, triglyceride-glucose index; TyG-BMI, TyG index corrected for BMI; TyG-WC, TyG index corrected for waist circumference; TG/HDL, plasma triglyceride/HDL cholesterol ratio.

### Receiver operating characteristic curve analysis to discriminate insulin sensitivity versus insulin resistance among girls and boys

**Figure 2:**
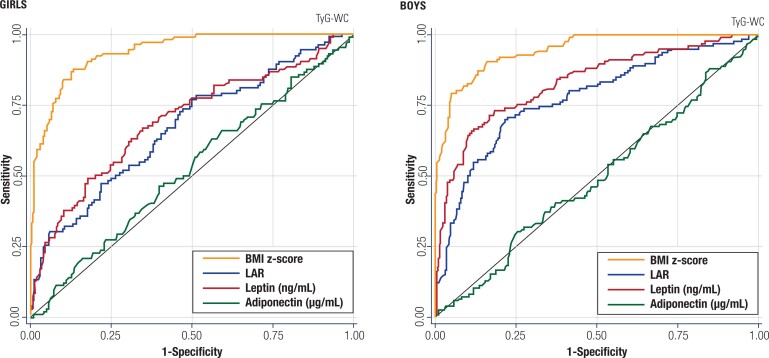
Receiver operating characteristic (ROC) curve analysis to discriminate insulin sensitivity versus resistance among 465 girls (left) and 503 boys (right) using the index triglyceride-glucose corrected for waist circumference (TyG-WC) as a binary classification variable. The TyG-WC 75th percentile was used to identify children with insulin resistance from those with insulin sensitivity. Plasma leptin, plasma adiponectin, leptin/adiponectin ratio (LAR), and BMI z-score were assessed using their areas under the ROC curve.

**Supplementary Figure 1:**
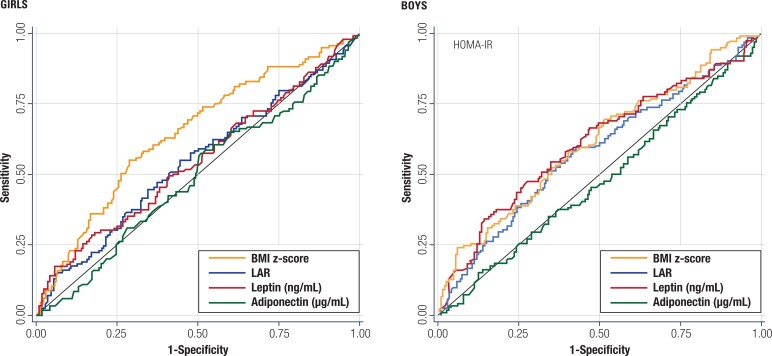
Receiver operating characteristic (ROC) curve analysis to discriminate insulin sensitivity versus resistance among 465 girls (left) and 503 boys (right) using the homeostasis model assessment of insulin resistance (HOMA-IR) as a binary classification variable. The HOMA-IR 75th percentile was used to identify children with insulin resistance from those with insulin sensitivity. Plasma leptin, plasma adiponectin, leptin/adiponectin ratio (LAR), and body mass index (BMI) z-score were assessed using their areas under the ROC curve.

As the TyG-WC index was the only insulin surrogate index associated with plasma leptin and LAR after adjustment for BMI, this variable was used to identify an insulin resistance status, defined as a TyG-WC index above the 75th percentile. Subsequently, we assessed, using ROC curves, the ability of plasma adipokines and LAR to discriminate insulin resistance status (Figure 2). As expected, the BMI z-score showed the highest area under the ROC curve and, therefore, had the best single performance in discriminating insulin resistance. The ability of LAR to discriminate insulin resistance was significant, with similar or even weaker performance compared with isolated plasma leptin. The area under the ROC curve for plasma adiponectin was not significantly different from 0.5 (null value). We also used the 75th percentile of the HOMA-IR as a classificatory variable of insulin resistance (Supplementary Figure 1). Again, the BMI z-score showed the highest area under the ROC curve and, therefore, had the best single performance in discriminating insulin resistance. Additionally, only in boys, both plasma leptin and LAR also showed a significant and similar capacity to identify insulin resistance from sensitivity (Supplementary Figure 1). Again, plasma adiponectin showed no discriminatory capacity to predict insulin resistance using the HOMA-IR cutoff points.

## DISCUSSION

The present study evaluated the performance of plasma leptin, plasma adiponectin, and LAR as surrogates of insulin resistance in prepubertal children. As expected, plasma leptin and LAR were significantly higher in children with obesity compared with those with normal weight (5). We also found that plasma leptin and LAR were significantly associated with all insulin surrogates assessed in the study (HOMA-IR, TyG, TyG-BMI, TyG-WC, and TG/HDL ratio). However, the TyG-WC index was the only insulin resistance surrogate that remained significantly associated with LAR after adjustment by BMI z-score. In the ROC curve analysis using insulin resistance as a categorized variable based on the TyG-WC index, the BMI z-score was the best single variable with the highest area under the ROC curve to identify children with insulin resistance from those with insulin sensitivity, followed by significant effects of plasma leptin and LAR. The area under the ROC curve for plasma adiponectin was not significantly different from the null value. The results indicate that most associations between LAR and insulin resistance indexes are determined by plasma leptin, which in turn is directly related to adiposity.

Circulating levels of leptin and adiponectin have been previously associated with insulin resistance (24,35). On the one hand, leptin has a positive association with adiposity, insulin resistance, and type 2 diabetes mellitus, while adiponectin has a negative relationship with adiposity and insulin resistance (36,37). In obesity, adipocytes secrete more leptin and less adiponectin, leading to the hypothesis that the LAR is a useful biomarker of adipocyte hypertrophy, insulin resistance, and cardiovascular risk (5). The association between LAR and insulin resistance was mainly driven by leptin over adiponectin in the present study, given that we found a weak association between plasma adiponectin and insulin resistance. In this context, increased leptin level, a common finding in obesity, is strongly associated with insulin resistance partially through increased adiposity, which suggests an overall decrease in sensitivity to leptin and insulin in obesity (38). Notably, the children in our study were prepubertal (Tanner 1) and had approximately 7 years of age (before the known rise in plasma insulin of adolescence), which may explain the nonsignificant results for the association between plasma adiponectin and insulin resistance.

Insulin resistance is an underlying factor in metabolic syndrome and is associated with cardiometabolic risk. However, there is no agreement on how to define and quantify insulin resistance, especially in children (39). In healthy adolescents, the physiologic decline in insulin sensitivity is accompanied by compensatory insulin secretion, which recovers once puberty is complete. Nevertheless, there is sufficient evidence showing that children with obesity do not recover baseline insulin sensitivity at the end of puberty. The onset of obesity-associated complications in adolescence, such as dysglycemia, usually coincides with puberty (40). Even in the absence of impairment of glucose tolerance, the decline in insulin sensitivity is associated with lower hepatic insulin clearance in obese adolescents, which may contribute to the decrease in beta-cell function over time (41–45). Hence, it is important to predict insulin resistance in children since its early detection would allow dietary/lifestyle interventions to avoid later metabolic impairment in adolescence and young adulthood (46).

It has been proposed that, in adolescents, leptin and LAR are associated with high insulin resistance independent of potential confounders (age, sex, pubertal stage, adherence to the Mediterranean diet, cardiorespiratory fitness, and body fat percentage) (47). These results are consistent with those of the present study, where we found a high risk of insulin resistance associated with leptin and LAR. In adults, a high correlation has been reported between LAR and a wide range of insulin resistance surrogates, from the simplest indexes, based on fasting plasma samples, to the more sophisticated ones, such as the M/I value in the hyperinsulinemic-euglycemic clamp (48). A significant correlation between LAR and insulin resistance indexes derived from oral or intravenous tolerance tests has also been found (5). In contrast, few studies have assessed the performance of LAR as a significant predictor of insulin resistance in prepubertal children (1,11), as evaluated in the present study. In a study of children aged 6-18 years, Frithioff-Bøjsøe and cols. (11) showed that those participants with overweight/obesity had 4 to 8 times higher odds of exhibiting insulin resistance if their LARs were in the upper quartile compared with those whose LAR ratio was in the lower quartile. In the present study, we evaluated LAR in the context of other insulin resistance surrogates based on fasting plasma samples (HOMA-IR, several TyG indexes, TG/HDL ratio) and specifically in prepubertal children. In contrast to other insulin resistance surrogates based on fasting blood samples, an advantage of leptin, adiponectin, and LAR over other insulin resistance surrogates may reside in their relative independence from previous dietary intake and the possibility of measuring them under nonfasting conditions (even assuming a small disturbance in the hormonal circadian rhythm).

In conclusion, in prepubertal Chilean children, plasma leptin and LAR were strongly associated with BMI z-scores and a wide range of insulin resistance surrogates commonly used in epidemiological studies. In contrast, plasma adiponectin was not significantly associated with adiposity or insulin resistance indexes. After adjustments for age, sex, and BMI z-score, plasma leptin and LAR remained strongly associated with the insulin resistance surrogate TyG-WC index. Associations between LAR and insulin resistance indexes are mainly driven by the effect of plasma leptin, which is also directly associated with increased adiposity.
